# Association between Serum Ferritin and Prognosis in Patients with Ischemic Heart Disease in Intensive Care Units

**DOI:** 10.3390/jcm12206547

**Published:** 2023-10-16

**Authors:** Shun Liu, Mingxian Chen, Liang Tang, Xuping Li, Shenghua Zhou

**Affiliations:** Department of Cardiovascular Medicine, The Second Xiangya Hospital, Central South University, Changsha 410011, China; nhdxhy@163.com (S.L.);

**Keywords:** marker, prognosis, ferritin, ischemic heart disease, MIMIC-IV database

## Abstract

Purpose: Recent years have seen a clear link established between elevated ferritin levels and COVID-19 prognosis. However, the impact of heightened ferritin levels on the prognosis of individuals with severe ischemic heart disease remains uncertain. Methods: We utilized the MIMIC IV database to identify a cohort of ischemic heart disease patients who underwent serum ferritin testing. We conducted regression analyses, employed the overlap propensity score weighting model, and utilized the restricted cubic splines model to comprehensively investigate the associations between serum ferritin levels and clinical outcomes. Results: Our cohort included 1173 patients with diagnosed ischemic heart disease, categorized into high and low serum ferritin groups. After meticulous adjustment for confounding factors in a fully adjusted model, the hazard ratios (HRs) for 90-day and 1-year mortality were 1.63 (95% CI: 1.27–2.09) and 1.49 (95% CI: 1.19–1.86), respectively, in the high-ferritin group compared to the low-ferritin group. Subsequent analyses with propensity score weighting confirmed these results. Remarkably, restricted cubic spline analysis revealed an almost linear relationship between log-transformed serum ferritin levels and the risk of both 90-day and 1-year all-cause mortality. Moreover, incorporating ferritin into conventional severity of illness scores significantly improved the area under the curve for both 90-day and 1-year mortality. Conclusions: This study provides compelling evidence regarding the prognostic significance of serum ferritin in predicting 90-day and one-year mortality rates among patients diagnosed with ischemic heart disease.

## 1. Introduction

In recent years, the escalation of global economic growth and enhancements in individuals’ living standards have contributed to ischemic heart disease (IHD) emerging as the primary cause of mortality among middle-aged and elderly populations [1]. Despite considerable advancements in risk factor management and treatment modalities, spearheaded by diligent medical professionals and scientists, patients afflicted with severe IHD still face significant mortality rates [2]. Hence, it becomes imperative to delve deeper into the pathogenesis of IHD and explore its prognostic implications to unravel novel treatment and intervention strategies.

In recent years, a substantial body of evidence has emphasized the association between elevated ferritin levels and the prognosis of COVID-19, triggering investigations into the underlying mechanisms [3,4,5]. The emergence of novel pathways such as ferritinophagy and ferroptosis has further heightened interest in exploring ferritin-related processes [6,7,8]. Serum ferritin, serving as an acute-phase protein and a crucial iron carrier, assumes a pivotal role in iron metabolism and exhibits intricate interactions with the oxidative stress response [9,10,11,12]. It is essential to emphasize that ferritin, as an acute-phase reactant protein, increases during the decompensated state of patients. This is noteworthy, as there is a considerable body of literature discussing the role of acute-phase indicators in these patients. These indicators hold significance in both acute and chronic phases of the disease [13,14,15]. Although several investigations have unveiled connections between serum ferritin and cardiovascular disease, a knowledge gap persists regarding the precise relationship between serum ferritin and severe IHD. Certain studies have indicated that elevated serum ferritin levels correlate with an augmented risk of developing and worsening coronary heart disease [16,17,18]. Nevertheless, conflicting outcomes have been reported in other studies, with some even failing to establish a definitive association [19,20].

As such, there is a compelling imperative to conduct a comprehensive investigation to delve into the intricate association between serum ferritin and severe IHD. Consequently, the primary aim of this study was to meticulously examine the potential correlation between serum ferritin levels and the prognostic implications for individuals afflicted with severe IHD. This study’s findings lay a crucial foundation for serum ferritin to serve as a reliable risk stratifier for critically ill patients.

## 2. Methods

### 2.1. The Data Source

The Medical Information Mart for Intensive Care-IV (MIMIC-IV) database, a comprehensive repository comprising more than 50,000 intensive care unit (ICU) admissions at Beth Israel Deaconess Medical Center in Boston, Massachusetts, spanning from 2008 to 2019, was utilized in this study [21]. Within the MIMIC-IV database, a wealth of data encompassing various elements such as demographic characteristics, vital signs, laboratory test results, and diagnoses coded according to the International Classification of Diseases and Ninth Revision (ICD-9) and International Classification of Diseases and Tenth Revision (ICD-10) systems is available. To access this valuable resource, one of the authors (ASY) obtained the necessary certification and extracted the required variables for the investigation (certification number: 46993042). It is worth noting that individual patient consent was not required, as the health information in the database underwent de-identification, ensuring the anonymity of patients.

### 2.2. Study Population

The present investigation encompassed a comprehensive cohort of patients who received a diagnosis of IHD according to ICD-9 and ICD-10 codes. Specifically, IHD was defined based on the first three digits of the diagnosis code, which included ‘410’, ‘412’, ‘414’, ‘I21’, ‘I22’, and ‘I25’. For the purpose of this study, data were exclusively obtained from the initial admission to the ICU for each patient. Inclusion criteria involved patients with available ferritin results within three days before ICU admission and prior to discharge. Within the selected cohort, individuals with ferritin levels surpassing 1000 ng/mL were classified as exhibiting iron overload group, as supported by previous research [22,23]. Those without iron overload comprised the control group. Exclusion criteria entailed patients with a survival duration of less than 24 h, individuals below 18 years of age, or cases with incomplete data amounting to less than 30%.

### 2.3. Data Extraction and Variables

Data extraction was performed utilizing structured query language with pgAdmin4 and PostgreSQL 13. Variables selection was based on a consensus approach, taking into consideration data availability, biological plausibility, and established associations. Initially, essential patient information, including age, gender, BMI, race, SAPSII score, SOFA score, Charlson comorbidity score, and GCS score, was extracted. Subsequently, variables encompassing vital signs, comorbidities, interventions, and laboratory findings were obtained. Finally, relevant clinical outcomes, including length of stay, in-hospital mortality, 90-day mortality, and 1-year mortality, were extracted. In cases where patients were admitted to the ICU within a 24 h window, only the most extreme values for each variable within that timeframe were included in this study, aligning with clinical experience. Further details regarding variables and their corresponding missing rates can be found in Supplementary Table S1. To address missing data within the MIMIC-IV database, the “mice” package in R was employed for imputation [24].

In our cohort, we encountered a notable absence of collected B-type natriuretic peptide (BNP) values for over half of the patients. Given the significance of BNP in assessing the condition of individuals with IHD, the high number of missing values posed a challenge. To address this, instead of directly utilizing BNP readings as a covariate, we employed the presence or absence of BNP values as the covariate in our analyses. Consequently, we incorporated a flag indicating whether BNP was recorded as a covariate in our models. Similarly, laboratory tests for troponin, creatinine kinase and creatinine kinase-MB were not ordered for more than half of the cohort. As a solution, we introduced flags representing whether these tests were obtained as covariates in our analysis.

### 2.4. Primary Outcome and Secondary Outcomes

The primary outcome of this study was the assessment of all-cause mortality at 90 days and 1 year. Secondary outcomes evaluated included in-hospital all-cause mortality and length of stay (LOS) in the ICU.

### 2.5. Statistical Methods

The baseline characteristics of the study population were summarized using descriptive statistics, presenting the mean and standard deviation (SD) for continuous variables, and the number and percentage for categorical variables. Group differences were assessed using appropriate statistical tests, such as the *t*-test, the Mann–Whitney U test, or the Kruskal–Wallis H test for continuous variables, and the chi-square test or Fisher’s exact test for categorical variables. The association between serum ferritin levels and the risk of 90-day and one-year mortality was explored using both univariable and multivariable Cox proportional hazards analyses. To ensure statistical efficiency and account for the available number of events and the total number of variables, it was important to streamline the selection of candidate variables. To identify the optimal set of variables for the multivariate Cox regression analysis, the forward-AIC (Akaike Information Criterion) stepwise method was employed. This iterative approach evaluates the AIC at each step, facilitating the selection of variables that best contribute to the final regression model. By balancing model fit and complexity, the forward-AIC stepwise method ensures that the selected variables effectively capture the relationship with the outcome of interest. This rigorous variable selection process enhances the accuracy and interpretability of the results in the final multivariate Cox regression model. Time-to-event distributions were assessed using the log-rank test and visualized using Kaplan–Meier curves. To account for confounding by indication a propensity score using the other variables was created, followed by use of overlap weighting to minimize the influence of extreme propensity scores on model output. The overlap propensity score weighting method, assigning weights to each patient based on the probability of being assigned to the opposite group [25], was applied to create a weighted cohort. The effectiveness of the overlap propensity score model in balancing the compared groups was evaluated by comparing the covariate imbalance between the original and weighted cohorts using standardized mean differences (SMDs). An SMDs < 0.2 for the measured covariate suggests an appropriate balance between groups. Cox regression was then performed on the weighted cohort to investigated associations between ferritin and the clinical outcomes, adjusting for variables that remained unbalanced between the groups with or without iron overload in the propensity score model. To assess potential unmeasured confounding effects, we calculated E-values, estimating the minimum strength of association (HR) required for an unmeasured confounder to explain the exposure–outcome association fully. Linear regression models assessed the relationship between two ferritin groups and ICU length of stay, while logistic regression models evaluated the connection between ferritin groups and in-hospital mortality. The predictive utility of ferritin, combined with traditional severity of illness scores, was evaluated for 90-day and 1-year all-cause mortality through receiver operating characteristic (ROC) analysis and area under curves (AUC) analysis.

### 2.6. Sensitivity Analyses and Exploratory Subgroup Analyses

To ensure the robustness and validity of our findings, we conducted model fitting using the original unimputed dataset, minimizing the potential impact of imputation. Furthermore, we divided the study patients into tertiles based on ferritin levels and performed pertinent regression analyses to elucidate the association between ferritin levels and all-cause mortality. Additionally, the relationship between serum ferritin levels and mortality in patients with IHD was further elucidated through the utilization of 3-knotted restricted cubic splines. Moreover, subgroup analyses were performed to investigate the association of ferritin levels with mortality within predefined subgroups based on age (above or below the median), gender, race (White or non-White), presence of congestive heart failure (CHF), presence of arrhythmias, presence of diabetes, and presence of renal disease. Cox proportional hazards models were employed to estimate hazard ratios (HRs) and 95% confidence intervals (CIs) for the risk within each subgroup. The interactions were rigorously evaluated using the Likelihood Ratio Test. All statistical analyses were conducted using R statistical software version 4.2.0 (R Project for Statistical Computing). A two-sided *p* value of less than 0.05 was considered statistically significant. These comprehensive analyses significantly contribute to the robustness and reliability of our study findings.

## 3. Results

### 3.1. Study Population and Baseline Characteristics

**Figure 1:**
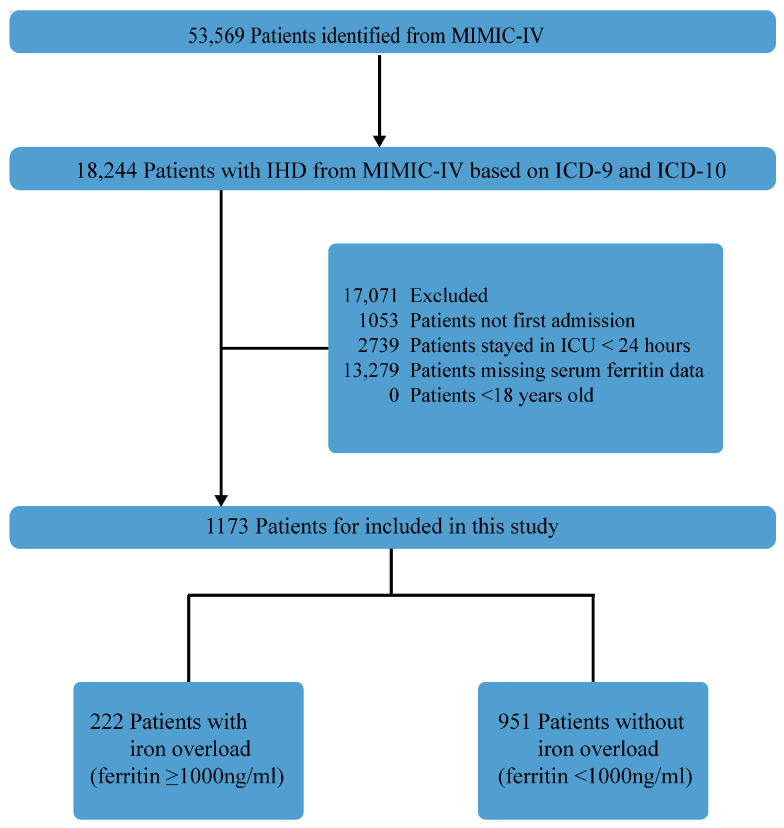
Flowchart illustrating the selection of patients from the MIMIC-IV database. MIMIC Medical Information Mart for Intensive Care.

The study cohort consisted of 1173 individuals diagnosed with IHD, identified from the MIMIC-IV database (Figure 1). Detailed baseline patient demographics, both pre- and post-propensity score weighting, are summarized in Table 1. Notably, the high-ferritin group exhibited significantly elevated serum iron levels compared to the control group, as presented in Table 1. Before overlap propensity score weighting, age, SAPAII score, SOFA score, heart rate, vasopressor use, certain comorbidities, and laboratory parameters showed differences between the two groups. However, after weighting, the SMDs of the variables between the two groups were less than 0.2. The similarity or balance of most covariates in the weighted cohorts between the iron overload and control groups is depicted in Supplementary Figure S1. In the weighting, a propensity score model was constructed using 42 covariates and the GBM method, with the individual contributions of covariates illustrated in Supplementary Figure S2. Subsequently, overlap weighting was applied based on the estimated propensity scores to minimize differences between the iron overload and control cohorts.

### 3.2. Primary Analysis

**Figure 2:**
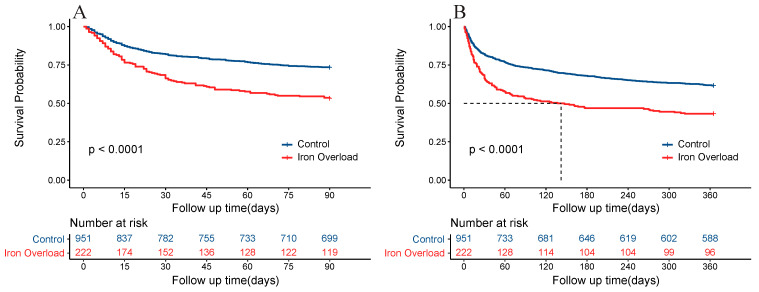
Kaplan–Meier survival curve of patients in the different serum ferritin level groups. (**A**) Serum ferritin and 90-day mortality. (**B**) Serum ferritin and 90-day mortality.

During the study period, the iron overload group experienced 104 deaths (46.8%) at 90 days and 126 deaths (56.8%) at 1 year. In contrast, the control group recorded 253 deaths (26.6%) at 90 days and 366 deaths (38.5%) at 1 year. Table 2 employed a comprehensive range of methodologies to thoroughly investigate the association between serum ferritin levels and mortality. In the unadjusted Cox analysis, the iron overload group exhibited hazard ratios (HRs) of 2.03 (95% CI: 1.62–2.55) and 1.79 (95% CI: 1.46–2.20) for 90-day and 1-year mortality, respectively, compared to the control group. After accounting for relevant factors through the adjusted models (including Model 2 and Model 3), the HRs for 90-day and 1-year mortality in the iron overload group remained significantly elevated relative to the control group. To enhance the robustness of these findings, an overlap weighting approach was employed to examine the relationship between ferritin and mortality. After applying overlap weighted data, the HRs for 90-day and 1-year mortality were 1.50 (95% CI: 1.17–1.93) and 1.35 (95% CI: 1.08–1.69), respectively, in the iron overload group compared to the control group. Additionally, Kaplan–Meier analysis plots demonstrated a significant disparity in the 90-day cumulative survival rate between ischemic heart disease patients with high ferritin and the control group (*p* < 0.0001, Figure 2A). Similar findings were observed in the one-year survival analysis (*p* < 0.0001, Figure 2B).

Receiver operating characteristic (ROC) analysis, coupled with area under the curve (AUC) analysis, was utilized to appraise the supplementary predictive capacity of ferritin when combined with established severity of illness scores for predicting 90-day and 1-year all-cause mortality. As illustrated in Supplementary Figure S3, the integration of ferritin with traditional severity of illness scores led to enhanced AUC values for 90-day all-cause mortality (Supplementary Figure S3A,B). This trend was consistently observed for 1-year all-cause mortality (Supplementary Figure S3C,D).

### 3.3. Secondary Analysis

During the designated study duration, patients belonging to the iron overload group demonstrated a mean length of stay of 8.91 days, accompanied by 69 in-hospital deaths (31.1%). In contrast, the control group exhibited a mean length of stay of 6.33 days, with 140 in-hospital deaths (14.7%). In Table 3, both adjusted and unadjusted linear regression analyses provided compelling evidence of a significantly higher in-hospital mortality rate in the iron overload group compared to the control group. Moreover, partially adjusted (Model 2) and unadjusted linear regression analyses indicated a significantly prolonged length of stay in the iron overload group in comparison to the control group.

### 3.4. Sensitivity Analysis

**Figure 3:**
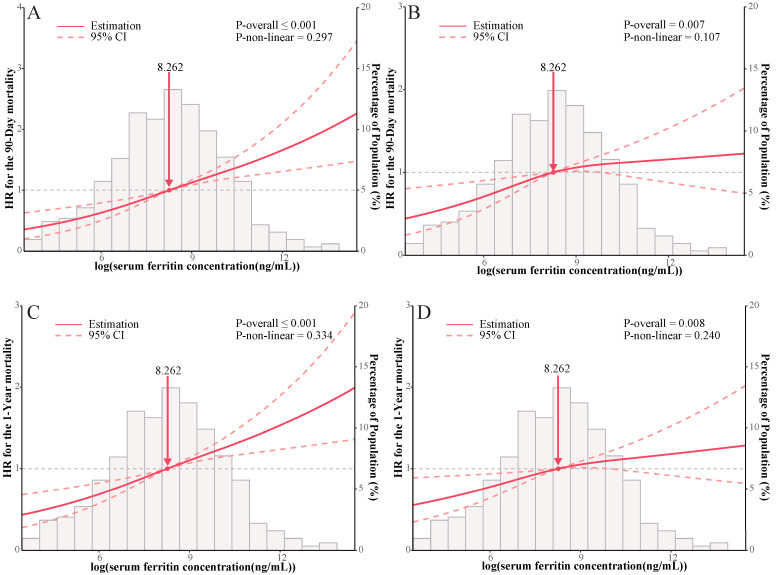
Association of log-transformed serum ferritin concentration and all-cause mortality in unadjusted and multivariable adjusted restricted cubic spline model. Hazard ratios are indicated by solid lines and 95% CI by long dashed lines. The reference values were set at 50th percentile, and the knots were set at 10th, 50th, and 90th percentiles of the log-transformed concentrations, respectively. serum ferritin concentrations after log-transformation are presented on the *X*-axis. The histograms represent the distributions of serum ferritin concentrations among the study population. (**A**) was unadjusted for serum ferritin concentration and 90-day all-cause mortality; (**B**) was adjusted for serum ferritin concentration and 90-day all-cause mortality. the adjusted variable aligns with the variable utilized in Model 3 (**C**) was unadjusted for serum ferritin concentration and 1-year all-cause mortality; (**D**) was adjusted for serum ferritin concentration and 1-year all-cause mortality. the adjusted variable aligns with the variable utilized in Model 3.

**Figure 4:**
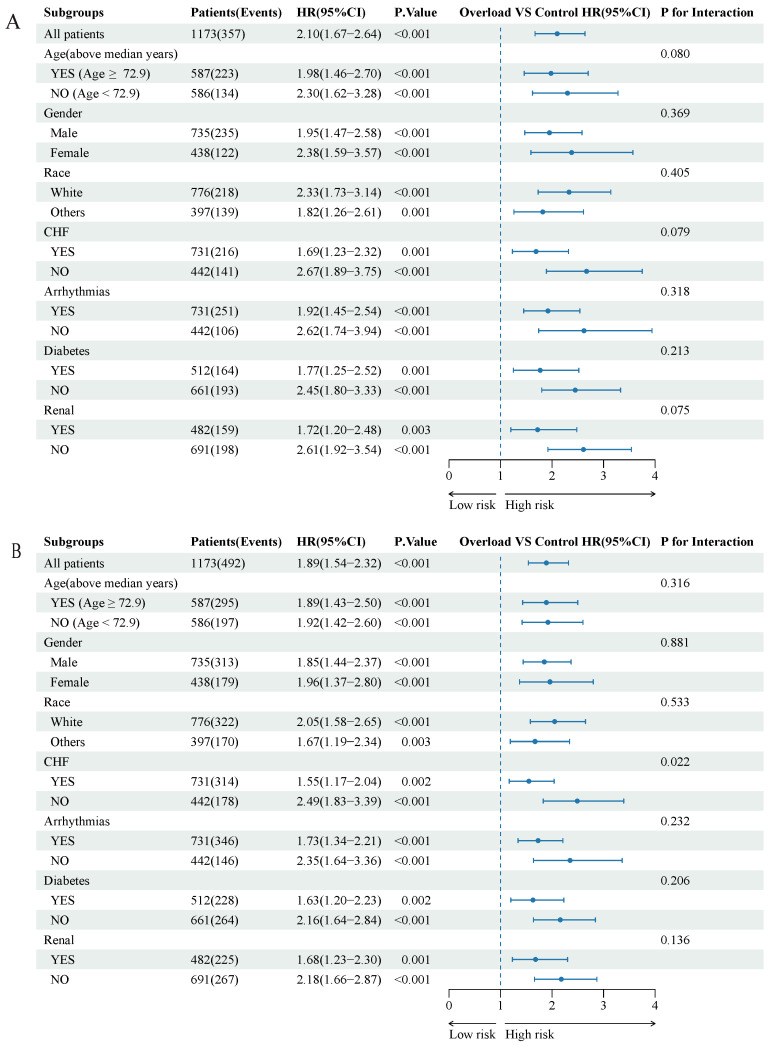
Forest plots of hazard ratios for the primary outcome in different subgroups. (**A**) display the hazard ratios (HRs) and corresponding 95% confidence intervals (CIs) for the relationship between serum ferritin concentration and 90-day all-cause mortality after adjusting for age, gender, and race. (**B**) display the hazard ratios (HRs) and corresponding 95% confidence intervals (CIs) for the relationship between serum ferritin concentration and 1-year all-cause mortality after adjusting for age, gender, and race. The interaction was tested using the likelihood ratio.

To ensure the robustness and reliability of our findings, we conducted model fitting using the original unimputed dataset, thereby minimizing potential biases associated with imputation. The consistency of our results was demonstrated in Supplementary Table S2, supporting the reliability of our findings. In Figure 3, we employed both unadjusted and multivariable adjusted restricted cubic splines to model and visualize the relationship between log-transformed serum ferritin concentration (as a continuous variable) and all-cause mortality in patients with ischemic heart diseases. This graphical representation revealed a progressive increase in the risk of death with higher serum ferritin concentrations, both at 90-day and one-year time points, surpassing the threshold of 307 ng/mL (all *p*-overall < 0.01). To explore potential variations in the relationship between serum ferritin and primary outcome across different subgroups, we performed stratified analyses based on age, gender, race, CHF, arrhythmias, diabetes, and renal disease. A comprehensive summary of these subgroup analyses is provided in Figure 4A,B. Notably, CHF exhibited an interaction with the association between serum ferritin and one-year all-cause mortality. Specifically, patients without CHF but with iron overload displayed an increased risk of death at one year. However, in other subgroups, the one-year mortality risk aligned with the overall risk, with no significant interactions observed. These findings contribute valuable insights into the relationship between serum ferritin levels and mortality outcomes, underscoring the importance of considering potential subgroup variations, particularly in the context of CHF.

To assess the potential impact of unmeasured confounding factors, we computed the E-value for the association between ferritin and 90-day mortality, utilizing the E-value methodology. This calculation yielded a value of 2.15. In practical terms, this implies that the observed hazard ratio (HR) of 1.40 could theoretically be accounted for by an unmeasured confounding variable that was intricately linked to both the treatment and the survival outcome, exerting a 2.15-fold influence on each of them. However, confounding of lesser strength would not have the same explanatory power. The E-value results for the ferritin-1-year mortality association yielded similar findings, indicating a consistent pattern of potential unmeasured confounding.

To enhance the robustness of our findings, we stratified the study participants into tertiles based on their ferritin levels. Supplementary Table S3 provides an overview of the baseline characteristics of patients, categorized by ferritin tertiles. The incidence of 90-day and 1-year all-cause mortality substantially increased with higher ferritin tertiles, peaking at 39.9% and 51.4%, respectively, in the high tertile group (Supplementary Table S3). In parallel, both length of stay and the risk of in-hospital mortality displayed upward trends with escalating ferritin tertiles. The cumulative risk of 90-day and 1-year all-cause mortality exhibited a significant upward trajectory in association with increasing ferritin levels. Importantly, this trend persisted even after meticulous adjustments for potential confounders in Model 3 (both *p* for trend < 0.01; Supplementary Table S4). After accounting for potential confounding factors, the hazard ratios (HRs) for 1-year all-cause mortality were 1.44 (1.13–1.84) and 1.29 (1.02–1.64) for the high and moderate groups, respectively, relative to the low-ferritin group (Supplementary Table S4). Analogous results were noted for the 90-day all-cause mortality rate, highlighting ferritin’s potential as a predictor of all-cause mortality. Additionally, our examination of the associations between ferritin and in-hospital death, as well as length of stay, revealed graded relationships of ferritin with both outcomes, with or without adjusting for potential confounders (Supplementary Table S5).

## 4. Discussion

This study presents compelling evidence regarding the prognostic value of serum ferritin in predicting 90-day and one-year mortality in critically ill patients with IHD admitted to the ICU. Our findings reveal a significant and linear association between log-transformed serum ferritin (≥307 ng/mL) and an increased incidence of mortality among IHD patients. Notably, this relationship remains robust even after meticulous adjustment for confounding factors, underscoring the independent relevance of serum ferritin with iron overload in predicting the risk of mortality. To mitigate potential biases and enhance the validity of our analysis, we employed overlap-weighting techniques, which effectively addressed differences in baseline variables between the iron overload and control groups. This approach serves as a valuable strategy to compensate for inherent limitations observed in retrospective cohort studies. Importantly, the results obtained from the overlap-weighted analysis align closely with those derived from the multifactor Cox analysis, reinforcing the credibility and reliability of our findings. Moreover, the inclusion of ferritin alongside baseline severity scores demonstrated an incremental effect on the predictive capability for all-cause mortality.

Serum ferritin serves as a widely recognized and essential indicator of iron status, playing a crucial role in the assessment of both iron deficiency and iron overload. While previous studies have reported associations between high levels of ferritin and the incidence of coronary heart disease, the prognostic implications of serum ferritin in relation to coronary artery disease have remained a subject of debate [26,27,28,29,30,31,32]. For instance, Feng et al. suggested that higher ferritin levels (>323 ng/mL) were associated with increased complications and mortality following myocardial infarction [23]. Similarly, Duarte et al. identified elevated ferritin levels (>316 ng/mL) as an independent prognostic factor in acute coronary syndromes [27]. In contrast, Gurgoze et al. concluded that serum ferritin levels were not associated with the prognosis of coronary artery disease [31,32]. Notably, these studies reported mean serum ferritin concentrations below 300 ng/mL in their cohorts, which significantly deviates from the baseline ferritin levels observed in our study. Furthermore, the limited number of endpoint events in these studies may have compromised the statistical power and effectiveness of their analyses. Additionally, these investigations primarily focused on patients with general coronary artery disease, with limited research specifically addressing the prognosis of serum ferritin in patients with severe ischemic heart disease.

Based on our meticulous multivariate regression analysis and subgroup analysis, we have established a robust and independent association between serum ferritin levels and the risk of 90-day and one-year all-cause mortality in patients diagnosed with IHD. Nevertheless, the precise underlying pathogenesis of this relationship remains incompletely elucidated. We propose several plausible explanations to shed light on these findings. Firstly, emerging studies have provided insights into the potential of iron overload to elicit chronic damage in affected organs, thereby leading to organ dysfunction [33,34,35,36,37]. Considering that the heart is a non-renewable organ, ischemia and hypoxia engender irreversible damage to cardiomyocytes. In parallel, iron overload exacerbates cellular death and diminishes the population of functional cardiomyocytes, ultimately impairing cardiac function and prognosis [30]. Secondly, as an exemplary acute-phase reactive protein, serum ferritin serves as a reflective indicator of systemic inflammatory responses, wherein elevated levels correspond to a more severe inflammatory state. This heightened inflammatory response contributes to augmented myocardial oxidative stress and injury, thereby exerting an impact on cardiac function and overall prognosis [35]. Remarkably, our sensitivity analysis, employing a sophisticated restricted cubic spline model, has unveiled a compelling linear relationship between log-transformed serum ferritin levels and the incidence of all-cause mortality. Furthermore, the observed serum ferritin concentration in these models closely approximates the concentration threshold recognized by an esteemed international panel of experts for metabolic hyperferritinemia [38]. In our subgroup analysis, we have discerned an interaction between congestive heart failure and serum ferritin in relation to one-year all-cause mortality. Specifically, critically ill patients with iron overload but without congestive heart failure exhibited a heightened risk of death at one year compared to critically ill patients without iron overload. This phenomenon may be attributed to the intrinsic severity of ICU patients’ underlying diseases, which inherently impairs their life expectancy irrespective of the coexistence of iron overload. Additionally, patients without heart failure, despite possessing a relatively prolonged life expectancy, may experience an exacerbation in the progression of pre-existing chronic diseases and manifest a worse prognosis when combined with iron overload. Lastly, given the rapid evolution of artificial intelligence, it is conceivable that future investigations will harness advanced big data analyses to unveil a more intricate relationship between ferritin and ischemic heart disease across its spectrum of severity [39].

Several limitations should be considered when interpreting the results of this study. Firstly, the retrospective design introduces inherent limitations. Although efforts were made to address confounding factors through overlap weighting to balance baseline characteristics between groups, the possibility of unmeasured confounders influencing the results cannot be completely ruled out. To further scrutinize this, we employed E-values, revealing a moderate level of robustness in our findings. Specifically, it suggests that an unmeasured confounding variable would require a substantial HR of 2.15 or higher, in relation to both exposure and outcome, to entirely account for the observed association. Secondly, our findings specifically pertain to patients with severe coronary artery disease and iron overload, and it remains unknown whether non-severe coronary artery disease with iron overload exhibits similar mortality patterns. Thirdly, the sample size of this study, particularly in subgroup analyses, was modest. The limitations associated with small sample size and potential selection bias should be acknowledged, and caution is advised when interpreting the results, particularly within specific subgroups. Future studies should prioritize larger sample sizes to provide more robust evidence supporting our findings. Lastly, the data collected in this study were limited to baseline measurements, and the follow-up period was relatively short. Therefore, changes in serum ferritin levels over time could not be evaluated. In summary, acknowledging the constraints of our retrospective study, future research should prioritize multicenter, randomized controlled trials with larger cohorts to address potential biases. These studies should encompass multifaceted interventions, extended follow-up periods and comprehensive assessments of ferritin and other inflammatory markers. This approach will enhance our understanding of the complex mechanisms linking ferritin to mortality in individuals with ischemic heart disease and refine our knowledge of critical threshold levels associated with adverse outcomes.

## 5. Conclusions

In summary, our study contributes to the growing body of evidence supporting the prognostic value of serum ferritin in predicting 90-day and one-year mortality in critically ill patients with IHD admitted to the ICU. These findings have important implications for the development of targeted interventions aimed at improving patient outcomes. Serum ferritin emerges as a potentially valuable tool for risk stratification and therapeutic management within the cohort of patients with IHD. However, it is important to note that further studies are necessary to validate these findings and to elucidate the impact of hyperferritin on prognosis in patients with common coronary artery disease. Continued research in this area will provide a deeper understanding of the clinical implications and potential interventions related to serum ferritin in ischemic heart disease.

## Figures and Tables

**Table 1:** Demographic Profiles of 1173 Patients with Ischemic Heart Disease before and after Propensity Score Weighting.

Variable Name	Unmatched			After Propensity Score Weighting		
	Control	Iron Overload	SMD	Control	Iron Overload	SMD
No.	951	222		951	222	
Log2(Ferritin) (mean (SD))	7.60 (1.52)	11.20 (1.43)	2.434	7.81 (1.45)	11.06 (1.25)	2.402
Demographic						
Age (mean (SD))	72.27 (12.51)	69.77 (12.45)	0.200	71.39 (12.25)	70.50 (12.54)	0.072
Gender (Female %)	366 (38.5)	72 (32.4)	0.127	46.4 (36.8)	39.3 (31.2)	0.118
BMI (mean (SD))	29.04 (7.32)	28.96 (8.28)	0.010	29.08 (7.30)	29.04 (8.56)	0.005
Race (White %)	640 (67.3)	136 (61.3)	0.126	82.7 (65.6)	76.4 (60.7)	0.103
Severity of Illness						
SAPSII Score (mean (SD))	40.04 (13.04)	45.93 (13.51)	0.444	43.60 (13.78)	44.14 (12.74)	0.041
SOFA Score (mean (SD))	6.05 (3.67)	8.34 (4.04)	0.594	7.31 (3.94)	7.77 (3.82)	0.117
Charlson Comorbidity Score (mean (SD))	7.52 (2.57)	8.00 (2.67)	0.185	7.78 (2.67)	7.90 (2.69)	0.043
GCS Score (mean (SD))	13.74 (2.63)	13.75 (2.37)	0.006	13.60 (2.76)	13.81 (2.23)	0.084
MBP (1st 24 h) (mean (SD))	56.49 (12.31)	54.56 (13.63)	0.148	55.18 (12.78)	55.19 (13.91)	0.001
Heart Rate (1st 24 h) (mean (SD))	102.49 (21.10)	110.92 (24.97)	0.365	105.48 (21.72)	108.92 (23.63)	0.152
Spo2 (mean (SD))	91.77 (6.59)	90.99 (6.52)	0.118	91.13 (7.92)	91.00 (6.16)	0.019
Comorbidities						
CHF (%)	611 (64.2)	120 (54.1)	0.209	77.9 (61.9)	69.7 (55.3)	0.133
Arrhythmias (%)	596 (62.7)	135 (60.8)	0.038	79.3 (62.9)	78.0 (62.0)	0.02
PVD (%)	205 (21.6)	43 (19.4)	0.054	26.4 (21.0)	23.4 (18.6)	0.059
Stroke (%)	138 (14.5)	35 (15.8)	0.035	20.2 (16.0)	21.0 (16.7)	0.019
COPD (%)	329 (34.6)	76 (34.2)	0.008	43.1 (34.2)	44.3 (35.2)	0.020
Diabetes (%)	420 (44.2)	92 (41.4)	0.055	56.0 (44.4)	52.4 (41.6)	0.058
Liver (%)	121 (12.7)	47 (21.2)	0.227	21.9 (17.3)	21.7 (17.2)	0.004
Renal (%)	392 (41.2)	90 (40.5)	0.014	53.2 (42.2)	50.2 (39.8)	0.048
Malignancy (%)	90 (9.5)	49 (22.1)	0.351	17.7 (14.0)	23.1 (18.4)	0.118
Interventions						
PCI (%)	93 (9.8)	19 (8.6)	0.042	11.7 (9.2)	10.8 (8.6)	0.023
CABG (%)	100 (10.5)	17 (7.7)	0.100	11.0 (8.7)	11.2 (8.9)	0.005
Sedative Use (1st 24 h) (%)	392 (41.2)	109 (49.1)	0.159	60.3 (47.9)	60.5 (48.0)	0.003
Vasopressor Use (1st 24 h) (%)	383 (40.3)	114 (51.4)	0.224	60.0 (47.7)	61.8 (49.1)	0.028
Mechanical Ventilation Use (1st 24 h) (%)	283 (29.8)	86 (38.7)	0.190	44.9 (35.7)	47.9 (38.0)	0.049
Laboratory Tests						
Lactate (mean (SD))	2.53 (2.08)	3.29 (3.18)	0.281	2.87 (2.44)	2.98 (2.65)	0.041
WBC (mean (SD))	13.96 (9.46)	15.27 (8.96)	0.143	14.97 (11.04)	15.07 (9.05)	0.010
Hemoglobin (mean (SD))	9.28 (2.08)	8.95 (2.26)	0.153	9.18 (2.10)	9.11 (2.24)	0.029
Platelet (mean (SD))	191.63 (100.62)	174.87 (110.71)	0.158	182.24 (110.48)	180.92 (105.51)	0.012
ALT (mean (SD))	109.17 (381.26)	310.63 (914.97)	0.287	180.37 (540.80)	213.87 (668.90)	0.055
AST (mean (SD))	166.09 (641.56)	547.70 (1732.55)	0.292	280.69 (898.54)	350.47 (1203.38)	0.066
Total Bilirubin (mean (SD))	1.16 (1.94)	2.69 (5.79)	0.353	1.57 (2.76)	1.88 (4.29)	0.085
BUN (mean (SD))	40.38 (29.23)	46.53 (33.98)	0.194	45.11 (32.85)	45.39 (34.09)	0.008
Creatinine (mean (SD))	2.05 (2.19)	2.98 (2.90)	0.362	2.56 (3.18)	2.75 (2.88)	0.065
Glucose (mean (SD))	184.07 (106.79)	202.19 (111.74)	0.166	194.39 (118.00)	202.39 (118.02)	0.068
Cholesterol (tested) (%)	160 (16.8)	32 (14.4)	0.066	19.1 (15.2)	19.0 (15.1)	0.002
Sodium (mean (SD))	139.39 (5.15)	139.88 (4.82)	0.097	139.45 (5.31)	139.83 (4.79)	0.075
Potassium (mean (SD))	4.72 (0.86)	4.82 (0.98)	0.110	4.81 (0.95)	4.83 (0.97)	0.018
Calcium (mean (SD))	8.43 (1.48)	8.37 (1.32)	0.041	8.41 (1.43)	8.38 (1.35)	0.019
Bicarbonate (mean (SD))	21.34 (5.20)	19.58 (5.65)	0.324	20.20 (5.32)	19.99 (5.45)	0.039
Chloride (mean (SD))	104.57 (6.77)	104.41 (6.55)	0.024	104.74 (6.98)	104.66 (6.35)	0.013
Anion Gap (mean (SD))	17.25 (4.84)	19.72 (6.19)	0.444	18.41 (5.34)	18.85 (5.50)	0.081
INR (mean (SD))	1.70 (1.13)	1.88 (1.49)	0.137	1.81 (1.36)	1.76 (1.25)	0.042
Iron (mean (SD))	46.70 (42.93)	70.23 (62.37)	0.440	47.41 (45.43)	64.21 (53.40)	0.339
Creatinine Kinase (tested) (%)	612 (64.4)	158 (71.2)	0.146	86.0 (68.2)	85.9 (68.2)	0.001
Creatinine Kinase-MB (tested) (%)	622 (65.4)	159 (71.6)	0.134	86.8 (68.9)	89.7 (71.2)	0.051
Troponin (tested) (%)	594 (62.5)	158 (71.2)	0.186	84.3 (66.9)	89.8 (71.3)	0.095
BNP (tested) (%)	278 (29.2)	60 (27.0)	0.049	32.5 (25.8)	31.6 (25.1)	0.016

For all continuous covariates, the mean values and standard deviations are reported. Abbreviations: BMI, body mass index; SAPSII score: simplified acute physiology score; SOFA score: sequential organ failure assessment score; GCS Score: glasgow coma scale score; MBP: mean blood pressure; CHF: congestive heart failure; PVD: peripheral vascular disease; COPD: chronic pulmonary disease; PCI: percutaneous coronary intervention; CABG: coronary artery bypass grafting; Spo2: partial pressure of oxygen; WBC: white blood cell; ALT: alanine aminotransferase; AST: aspartate aminotransferase; BUN: blood urea nitrogen; INR: international normalized ratio; BNP: B-type natriuretic peptide.

**Table 2:** Elucidating the Association between Different Serum Ferritin Level Groups and Primary Outcomes.

Primary Analysis				
90-Day Mortality				
Cox Regression	HR	CIL	CIU	*p* value
Model 1	2.03	1.62	2.55	<0.001
Model 2	2.10	1.67	2.64	<0.001
Model 3	1.63	1.27	2.09	<0.001
Cox Analysis after Weighting	1.50	1.17	1.93	0.001
1-Year Mortality				
Cox Regression	HR	CIL	CIU	*p* value
Model 1	1.79	1.46	2.20	<0.001
Model 2	1.89	1.54	2.32	<0.001
Model 3	1.49	1.19	1.86	<0.001
Cox Analysis after Weighting	1.35	1.08	1.69	0.008

Model 1: unadjusted; Model 2: adjusted for age, gender, race; Model 3: adjusted for age, gender, race, SAPSII score, SOFA score, Charlson comorbidity score, CHF, arrhythmias, stroke, COPD, liver, malignancy, CABG, mechanical ventilation use, Spo2, lactate, WBC, hemoglobin, platelet, total bilirubin, AST, BUN, creatinine, chloride, and INR. Abbreviations: HR hazard ratio, CIL confidence interval lower; CIU: confidence interval upper.

**Table 3:** Elucidating the Association between Different Serum Ferritin Level Groups and Secondary Outcomes.

Secondary Analysis				
Length of Stay in ICU				
Linear Regression	Coef.	S.E.	t-value	*p* value
Model 1	2.58	0.65	3.99	<0.001
Model 2	2.42	0.65	3.72	<0.001
Model 3	0.96	0.61	1.58	0.115
In-Hospital Mortality				
Logistic Regression	OR	CIL	CIU	*p* value
Model 1	2.61	1.86	3.65	<0.001
Model 2	2.70	1.91	3.80	<0.001
Model 3	1.88	1.24	2.81	0.002

Model 1: unadjusted; Model 2: adjusted for age, gender, race; Model 3: adjusted for age, gender, race, SAPSII score, SOFA score, Charlson comorbidity score, CHF, arrhythmias, stroke, COPD, liver, malignancy, CABG, mechanical ventilation use, Spo2, lactate, WBC, hemoglobin, platelet, total bilirubin, AST, BUN, creatinine, chloride, INR. Abbreviations: Coef. coefficient, S.E standard error, OR odds ratio, CIL confidence interval lower; CIU: confidence interval upper.

## Data Availability

The datasets produced and analyzed during the present study are obtainable from the corresponding author upon reasonable request.

## Supplementary Materials

The following supporting information can be downloaded at: https://www.mdpi.com/article/10.3390/jcm12206547/s1, Figure S1: Comparing the covariate between the original and adjusted (weighted) cohorts using standardized mean differences. Figure S2: Relative influence factor of covariates. Figure S3: Area under ROC curve of Ferritin combined with various scores for 90-day mortality and 1-year mortality; Table S1: Patient Demographic Characteristics and Proportion of Missing Data at Baseline before and after Imputation. Table S2: Elucidating the Association between Different Serum Ferritin Level Groups and All-Cause Mortality. Table S3: Demographic Profiles of 1173 Patients with Ischemic Heart Disease by Tertiles of Ferritin. Table S4: Elucidating the Association Between Different Serum Ferritin Tertiles and Primary Outcomes. Table S5: Elucidating the Association Between Different Serum Ferritin Tertiles and Secondary Outcomes.

## Abbreviations

ICU	Intensive care unit
MIMIC-IV	Medical Information Mart for Intensive Care IV
ICD	International Classification of Diseases
IHD	ischemic heart disease
AIC	Akaike Information Criterion
SMDs	standardized mean differences
HR	hazard ratio
CIs	confidence intervals
BMI	body mass index
SAPSII Score	simplified acute physiology score
SOFA Score	sequential organ failure assessment score
GCS Score	glasgow coma scale score
MBP	mean blood pressure
CHF	congestive heart failure
PVD	peripheral vascular disease
COPD	chronic pulmonary disease
PCI	percutaneous coronary intervention
CABG	coronary artery bypass grafting
Spo2	partial pressure of oxygen
WBC	white blood cell
ALT	alanine aminotransferase
AST	aspartate aminotransferase
BUN	blood urea nitrogen
INR	international normalized ratio
BNP	B-type natriuretic peptide
