# Supplementary material for: Association between Serum Ferritin and Prognosis in Patients with Ischemic Heart Disease in Intensive Care Units
Source: J Clin Med. 2023 Oct 16;12(20):6547. doi: 10.3390/jcm12206547 (PMC10607098; doi:10.3390/jcm12206547)
Supplement: Supplementary file 1 [file jcm-12-06547-s001.zip › jcm-2639282-supplementary.pdf]

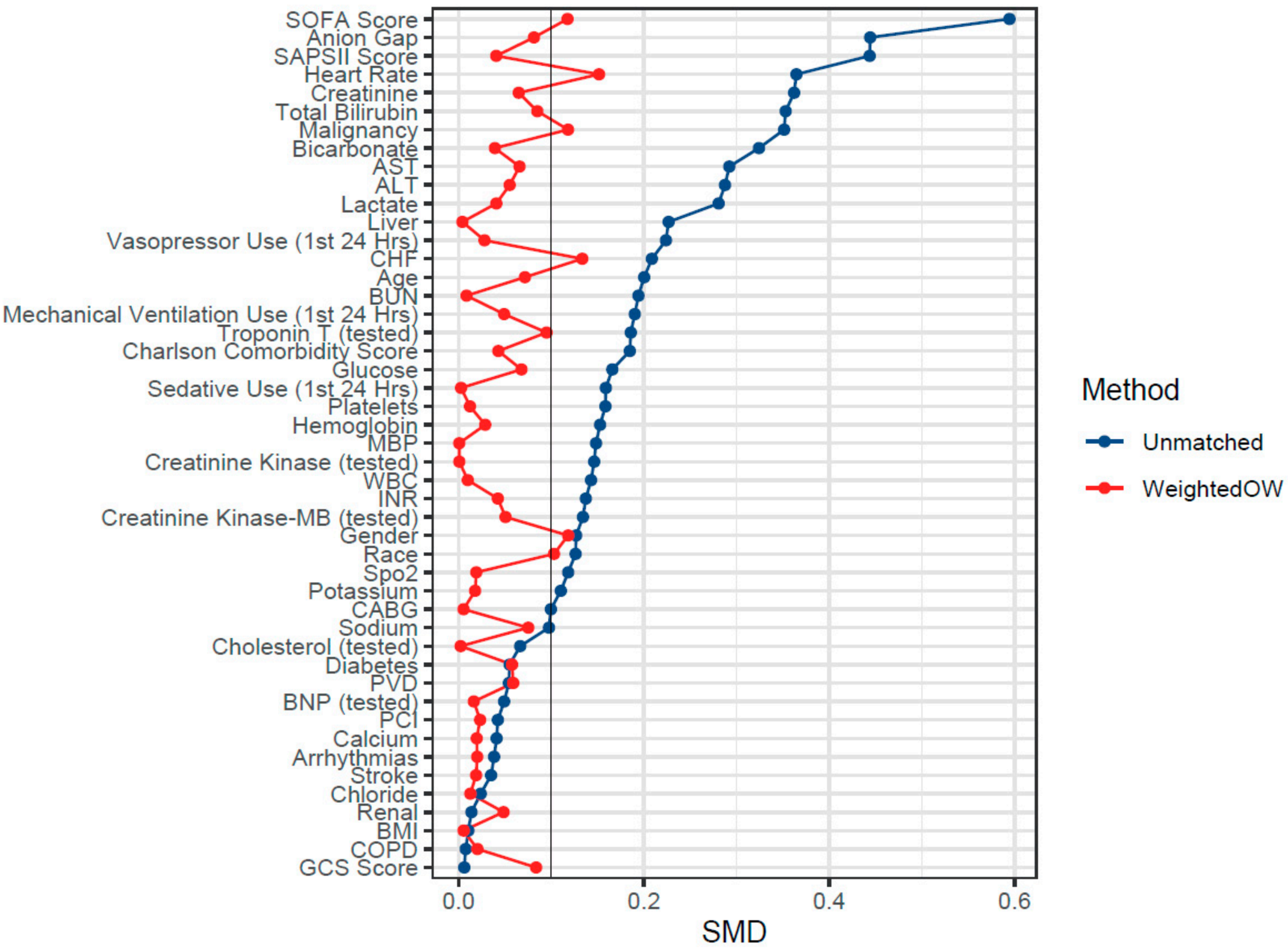

Supplementary Figure S1. comparing the covariate between the original and adjusted (weighted) cohorts using standardized mean differences.

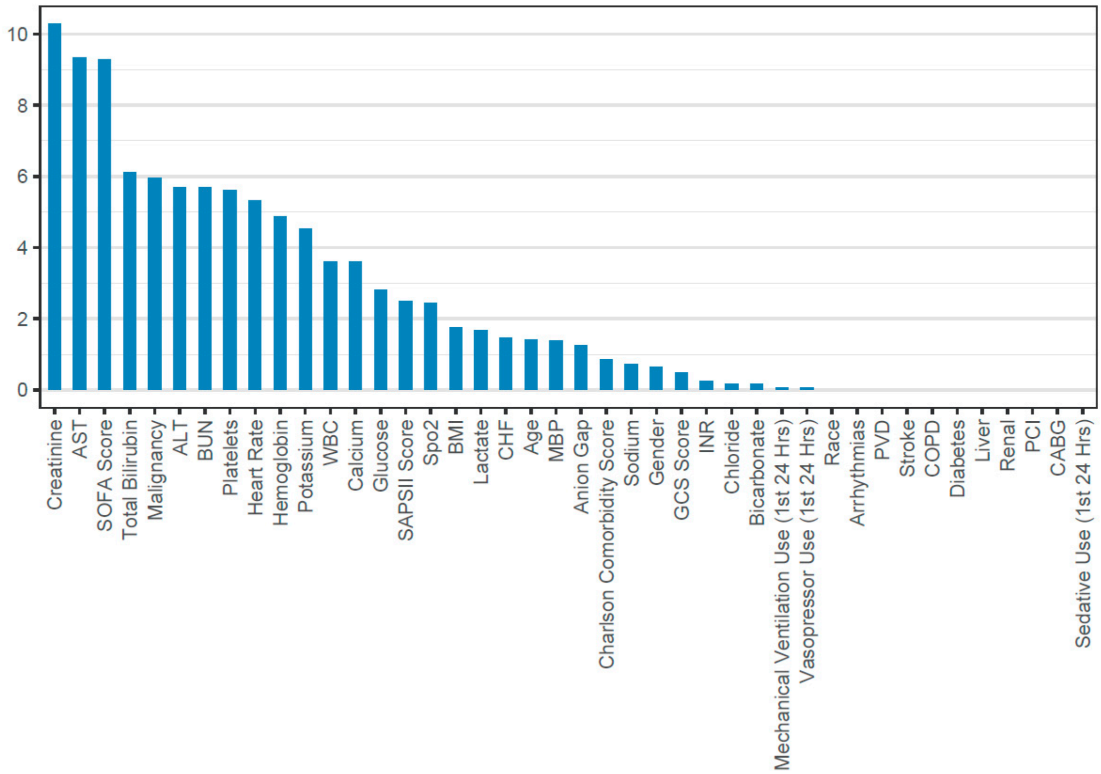

Supplementary Figure S2. Relative influence factor of covariates. The relative influence factor measures how discriminative the 42 covariates of the propensity score model is when predicting the likelihood of ferritin with Iron overload.

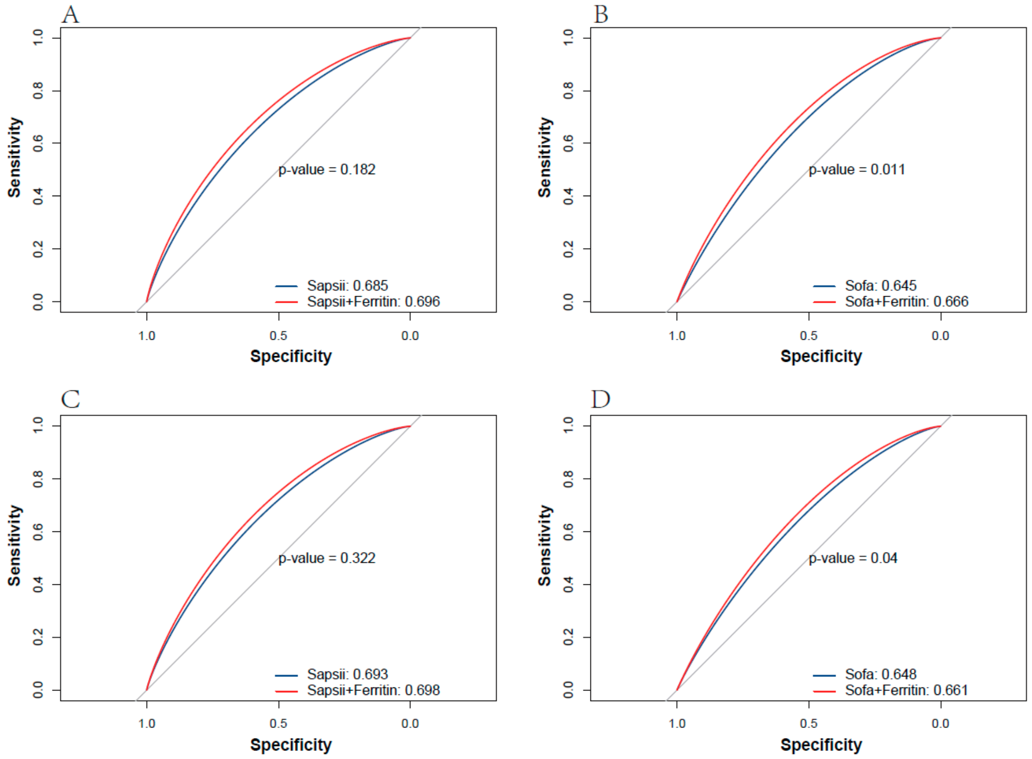

Supplementary Figure S3. Area under ROC curve of Ferritin combined with various scores for 90-day mortality and 1-year mortality. **A** Discrimination of 90-day mortality using Sapsii scores with and without Ferritin. **B** Discrimination of 90-day mortality using Sofa scores with and without Ferritin. **C** Discrimination of 1-year mortality using Sapsii scores with and without Ferritin. **D** Discrimination of 1-year mortality using Sofa scores with and without Ferritin.

Supplementary Table S1. Patient Demographic Characteristics and Proportion of Missing Data at Baseline before and after Imputation.

	Raw Data	Imputed Data	P	Missing.percent (100%)
NO.	1173	1173		
Age (mean (SD))	71.8 (12.54)	71.8 (12.54)	1.00	0
Gender (female)(%)	438 (62.66%)	438 (62.66%)	1.00	0
BMI (mean (SD))	29.23 (7.56)	29.29 (7.58)	0.85	28.9
Race (White) (%)	776 (66.16%)	776 (66.16%)	1.00	0
SAPSII Score (mean (SD))	41.16 (13.32)	41.16 (13.32)	1.00	0
SOFA Score (mean (SD))	6.48 (3.85)	6.48 (3.85)	1.00	0
Charlson Comorbidity Score (mean(SD))	7.61 (2.59)	7.61 (2.59)	1.00	0
GCS Score (mean (SD))	13.74 (2.59)	13.74 (2.58)	0.97	0.34
MBP(1st 24h) (mean (SD))	56.11 (12.6)	56.14 (12.61)	0.93	0.26
Heart Rate(1st 24h) (mean (SD))	104.08 (22.14)	104.07 (22.12)	1.00	0.26
CHF (%)	731 (62.32%)	731 (62.32%)	1.00	0
Arrhythmias (%)	731 (62.32%)	731 (62.32%)	1.00	0
PVD (%)	248 (21.14%)	248 (21.14%)	1.00	0
Stroke (%)	173 (14.75%)	173 (14.75%)	1.00	0
COPD (%)	405 (34.53%)	405 (34.53%)	1.00	0
Diabetes (%)	512 (43.65%)	512 (43.65%)	1.00	0
Liver (%)	168 (14.32%)	168 (14.32%)	1.00	0
Renal (%)	482 (41.09%)	482 (41.09%)	1.00	0
Malignancy (%)	139 (11.85%)	139 (11.85%)	1.00	0
PCI (%)	112 (9.55%)	112 (9.55%)	1.00	0
CABG (%)	117 (9.97%)	117 (9.97%)	1.00	0
Sedative Use (1st 24h) (%)	501 (42.71%)	501 (42.71%)	1.00	0
Vasoperessor Use (1st 24h) (%)	497 (42.37%)	497 (42.37%)	1.00	0
Mechanical Ventilation Use (1st 24h) (%)	369 (31.46%)	369 (31.46%)	1.00	0
Spo2 (mean (SD))	91.62 (6.58)	91.62 (6.58)	1.00	0
Lactate (mean (SD))	2.8 (2.47)	2.68 (2.36)	0.17	14.75
WBC (mean (SD))	14.21 (9.39)	14.21 (9.38)	0.97	0.17
Hemoglobin (mean (SD))	9.21 (2.11)	9.21 (2.12)	1.00	0.17
Platelet (mean (SD))	188.34 (102.79)	188.56 (102.84)	0.95	0.17
ALT (mean (SD))	158.19 (564.53)	146.89 (536.06)	0.55	14.58
AST (mean (SD))	257.13 (1022.43)	243.02 (1020.92)	0.54	13.9
Total Bilirubin (mean (SD))	1.49 (3.23)	1.45 (3.1)	0.81	12.96
BUN (mean (SD))	41.5 (30.25)	41.57 (30.33)	0.97	0.09
Creatinine (mean (SD))	2.22 (2.37)	2.23 (2.37)	0.98	0.09
Glucose (mean (SD))	187.5 (107.93)	187.5 (107.93)	1.00	0
Cholesterol(tested) (%)	192 (16.37%)	192 (16.37%)	1.00	0
Sodium (mean (SD))	139.48 (5.09)	139.48 (5.09)	1.00	0
Potassium (mean (SD))	4.74 (0.88)	4.74 (0.88)	1.00	0
Calcium (mean (SD))	8.41 (1.45)	8.41 (1.44)	0.94	0.6
Bicarbonate (mean (SD))	21 (5.33)	20.99 (5.34)	0.97	0.09
Chloride (mean (SD))	104.54 (6.73)	104.54 (6.73)	1.00	0

Anion Gap (mean (SD))	17.71 (5.19)	17.71 (5.19)	0.98	0.09
INR (mean (SD))	1.74 (1.21)	1.73 (1.2)	0.88	4.52
Iron (mean (SD))	51.33 (47.82)	52.13 (48.7)	0.86	21.48
Creatinine Kinase(tested) (%)	770 (65.64%)	770 (65.64%)	1.00	0
Creatinine Kinase-MB(tested) (%)	781 (66.58%)	781 (66.58%)	1.00	0
Troponin(tested) (%)	752 (64.11%)	752 (64.11%)	1.00	0
BNP(tested) (%)	338 (28.82%)	338 (28.82%)	1.00	0
Survival day (mean (SD))	326.31 (435.52)	326.31 (435.52)	1.00	0
Length Of Stay (mean (SD))	6.82 (8.74)	6.82 (8.74)	1.00	0
90_Day Mortality (%)	357 (30.43%)	357 (30.43%)	1.00	0
1_Year Mortality(%)	492 (41.94%)	492 (41.94%)	1.00	0
In-Hospital Death	209 (17.82%)	209 (17.82%)	1.00	0

Supplementary Table S2. Elucidating the Association between Different Serum Ferritin Level Groups and All-Cause Mortality.

**Rawdata Analysis**				
**90-Day Mortality**				
**Cox Regression**	**HR**	**CIL**	**CIU**	**P-value**
**Model 1**	2.03	1.62	2.55	<0.001
**Model 2**	2.10	1.67	2.64	<0.001
**Model 3**	1.61	1.23	2.11	<0.001
**1-Year Mortality**				
**Cox Regression**	**HR**	**CIL**	**CIU**	**P-value**
**Model 1**	1.79	1.46	2.20	<0.001
**Model 2**	1.89	1.54	2.32	<0.001
**Model 3**	1.49	1.17	1.90	<0.001

Model 1: unadjusted **Model 2**: adjusted for age, gender, race; **Model 3**: adjusted for age, gender, race, SAPSII score, SOFA score, charlson comorbidity score, CHF, arrhythmias, stroke, COPD, liver, malignancy, CABG, mechanical ventilation use, Spo2, lactate, WBC, hemoglobin, platelet, total bilirubin, AST, BUN, creatinine, chloride, INR; Abbreviations: HR hazard ratio, CIL confidence interval lower; CIU: confidence interval upper.

Supplementary Table S3. Demographic Profiles of 1173 Patients with Ischemic Heart Disease by tertiles of Ferritin

	ALL<br>N=1173	Low<br>N=391	Moderate<br>N=391	High<br>N=391	*P* value
Ferritin	307 [129;729]	85.0 [44.0;129]	307 [240;406]	1097 [730;1718]	<0.001
**Demographic**					
Age (mean (SD)	71.8 (12.5)	73.0 (12.7)	71.7 (13.0)	70.6 (11.8)	0.026
Gender (Female %)	438 (37.3%)	176 (45.0%)	131 (33.5%)	131 (33.5%)	0.001
BMI (mean (SD))	29.0 (7.51)	29.0 (7.19)	29.2 (7.43)	28.9 (7.90)	0.805
Race (White %)	776 (66.2%)	262 (67.0%)	257 (65.7%)	257 (65.7%)	0.909
**Severity of Illness**					
SAPSII Score (mean (SD))	41.2 (13.3)	37.5 (11.9)	40.3 (12.5)	45.6 (14.2)	<0.001
SOFA Score (mean (SD))	6.48 (3.85)	5.23 (3.20)	6.14 (3.59)	8.07 (4.14)	<0.001
Charlson Comorbidity Score (mean (SD))	7.61 (2.59)	7.47 (2.53)	7.50 (2.59)	7.87 (2.65)	0.054
GCS Score (mean (SD))	13.7 (2.58)	13.9 (2.26)	13.7 (2.65)	13.6 (2.80)	0.111
MBP (1st 24h) (mean (SD))	56.1 (12.6)	57.1 (11.7)	55.6 (12.1)	55.7 (13.8)	0.198
Heart Rate (1st 24h) (mean (SD))	104 (22.1)	102 (20.1)	103 (22.3)	108 (23.4)	<0.001
Spo2 (mean (SD))	91.6 (6.58)	91.8 (5.99)	92.0 (6.49)	91.1 (7.19)	0.129
**Comorbidities**					
CHF (%)	731 (62.3%)	256 (65.5%)	250 (63.9%)	225 (57.5%)	0.053
Arrhythmias (%)	731 (62.3%)	243 (62.1%)	239 (61.1%)	249 (63.7%)	0.759
PVD (%)	248 (21.1%)	80 (20.5%)	78 (19.9%)	90 (23.0%)	0.530
Stroke (%)	173 (14.7%)	58 (14.8%)	56 (14.3%)	59 (15.1%)	0.954
COPD (%)	405 (34.5%)	149 (38.1%)	125 (32.0%)	131 (33.5%)	0.171
Diabetes (%)	512 (43.6%)	177 (45.3%)	178 (45.5%)	157 (40.2%)	0.232
Liver (%)	168 (14.3%)	45 (11.5%)	56 (14.3%)	67 (17.1%)	0.080
Renal (%)	482 (41.1%)	153 (39.1%)	167 (42.7%)	162 (41.4%)	0.588
Malignancy (%)	139 (11.8%)	31 (7.93%)	38 (9.72%)	70 (17.9%)	<0.001
**Interventions**					
PCI (%)	112 (9.55%)	41 (10.5%)	40 (10.2%)	31 (7.93%)	0.407
CABG (%)	117 (9.97%)	48 (12.3%)	37 (9.46%)	32 (8.18%)	0.148
Sedative Use (1st 24h) (%)	501 (42.7%)	140 (35.8%)	168 (43.0%)	193 (49.4%)	0.001
Vasopressor Use (1st 24h) (%)	497 (42.4%)	153 (39.1%)	150 (38.4%)	194 (49.6%)	0.002
Mechanical Ventilation Use (1st 24h)	369 (31.5%)	102 (26.1%)	114 (29.2%)	153 (39.1%)	<0.001
**Laboratory Tests**					
Lactate (mean (SD))	2.67 (2.35)	2.51 (1.88)	2.44 (2.00)	3.08 (2.96)	<0.001
WBC (mean (SD))	14.2 (9.38)	12.9 (6.63)	14.0 (8.17)	15.8 (12.2)	<0.001

Hemoglobin (mean (SD))	9.22 (2.12)	9.22 (2.07)	9.36 (2.12)	9.09 (2.17)	0.206
Platelet (mean (SD))	188 (103)	193 (96.6)	195 (101)	178 (110)	0.036
ALT (mean (SD))	147 (531)	111 (435)	97.9 (294)	233 (749)	<0.001
AST (mean (SD))	238 (960)	171 (698)	135 (426)	409 (1435)	<0.001
Total Bilirubin (mean (SD))	1.45 (3.12)	0.91 (1.11)	1.27 (2.12)	2.17 (4.76)	<0.001
BUN (mean (SD))	41.5 (30.3)	37.1 (27.4)	42.9 (30.1)	44.6 (32.7)	0.001
Creatinine (mean (SD))	2.23 (2.37)	1.70 (1.40)	2.21 (2.03)	2.78 (3.20)	<0.001
Glucose (mean (SD))	187 (108)	176 (82.9)	188 (119)	198 (117)	0.018
Cholesterol(tested) (mean (SD))	192 (16.4%)	73 (18.7%)	64 (16.4%)	55 (14.1%)	0.220
Sodium (mean (SD))	139 (5.09)	140 (5.07)	139 (5.41)	140 (4.76)	0.083
Potassium (mean (SD))	4.74 (0.88)	4.71 (0.90)	4.75 (0.88)	4.76 (0.88)	0.660
Calcium (mean (SD))	8.42 (1.45)	8.52 (1.42)	8.40 (1.46)	8.33 (1.46)	0.195
Bicarbonate (mean (SD))	21.0 (5.33)	22.3 (5.12)	20.9 (4.98)	19.7 (5.57)	<0.001
Chloride (mean (SD))	105 (6.73)	105 (6.74)	105 (6.82)	105 (6.64)	0.994
Anion Gap (mean (SD))	17.7 (5.21)	16.5 (4.74)	17.6 (4.70)	19.1 (5.80)	<0.001
INR (mean (SD))	1.73 (1.21)	1.67 (1.07)	1.75 (1.24)	1.78 (1.30)	0.418
Iron (mean (SD))	51.2 (48.1)	48.1 (45.8)	45.5 (40.7)	59.8 (55.6)	<0.001
Creatinine Kinase(tested) (%)	770 (65.6%)	239 (61.1%)	259 (66.2%)	272 (69.6%)	0.044
Creatinine Kinase-MB (tested) (%)	781 (66.6%)	245 (62.7%)	262 (67.0%)	274 (70.1%)	0.087
Troponin(tested) (%)	752 (64.1%)	233 (59.6%)	247 (63.2%)	272 (69.6%)	0.013
BNP (tested) (%)	338 (28.8%)	132 (33.8%)	106 (27.1%)	100 (25.6%)	0.027
Length of Stay	6.82 (8.74)	4.77 (5.93)	6.44 (7.72)	9.23 (11.2)	<0.001
90-Day Mortality	357 (30.4%)	86 (22.0%)	115 (29.4%)	156 (39.9%)	<0.001
1-Year Mortality	492 (41.9%)	126 (32.2%)	165 (42.2%)	201 (51.4%)	<0.001
In-Hospital Mortality	209 (17.8%)	42 (10.7%)	62 (15.9%)	105 (26.9%)	<0.001

Data are presented as mean±SD or as n (%)

The patients were stratified by ferritin tertiles: low (<174, reference group), moderate (174–543), high (>544)

P-value for the test of the difference across tertiles of ferritin using the chi-square test (categorical variables), analysis of variance (continuous variables), or Kruskal–Wallis H test (nonparametric comparisons)

Abbreviations: BMI, body mass index; SAPSII Score: simplified acute physiology score; SOFA Score: sequential organ failure assessment score; GCS Score: glasgow coma scale score ; MBP: mean blood pressure; CHF: congestive heart failure; PVD: peripheral vascular disease; COPD: chronic pulmonary disease; PCI: percutaneous coronary intervention; CABG: coronary artery bypass grafting; Spo2: partial pressure of oxygen; WBC: white blood cell; ALT: alanine aminotransferase; AST: aspartate aminotransferase; BUN: blood urea nitrogen; INR: international normalized ratio; BNP: B-type natriuretic peptide.

Supplementary Table S4. Elucidating the Association Between Different Serum Ferritin Tertiles and Primary Outcomes

		Model 1		Model 2		Model 3	
**Primary Analysis**	**Events/No.**	**HR (95% CI)**	**P value**	**HR (95% CI)**	**P value**	**HR (95% CI)**	**P value**
**90-Day Mortality**							
Low (<174)	86(391)	ref		ref		ref	
Moderate (175~543)	115(391)	1.39(1.05-1.84)	0.020	1.42(1.07-1.88)	0.014	1.25(0.94-1.67)	0.127
High (>544)	156(391)	2.04(1.57-2.66)	<0.001	2.13(1.63-2.78)	<0.001	1.52(1.13-2.03)	0.005
P for trend			<0.001		<0.001		0.005
**1-Year Mortality**							
Low (<174)	126(391)	ref		ref		ref	
Moderate (175~543)	165(391)	1.40(1.11-1.76)	0.005	1.44(1.14-1.82)	0.002	1.29(1.02-1.64)	0.035
High (>544)	201(391)	1.89(1.51-2.36)	<0.001	2.00(1.60-2.50)	<0.001	1.44(1.13-1.84)	0.004
P for trend			<0.001		<0.001		0.004

Model 1: unadjusted **Model 2**: adjusted for age, gender, race; **Model 3**: adjusted for age, gender, race, SAPSII score, SOFA score, charlson comorbidity score, CHF, arrhythmias, stroke, COPD, liver, malignancy, CABG, mechanical ventilation use, Spo2, lactate, WBC, hemoglobin, platelet, total bilirubin, AST, BUN, creatinine, chloride, INR; Abbreviations: Coef. coefficient;

Supplementary Table S5. Elucidating the Association Between Different Serum Ferritin Tertiles and Secondary Outcomes.

**Primary Analysis**	**Model 1**		**Model 2**		**Model 3**	
**Length of Stay in ICU**						
**Linear Gegression**	**Coef**	**P value**	**Coef**	**P value**	**Coef**	**P value**
Low (<174)	ref		ref		ref	
Moderate (175~543)	1.67	0.006	1.58	0.010	1.11	0.043
High (>544)	4.45	<0.001	4.31	<0.001	2.46	<0.001
**In-Hospital Mortality**						
**Logistic Gegression**	**HR (95% CI)**	**P value**	**HR (95% CI)**	**P value**	**HR (95% CI)**	**P value**
Low (<174)	ref		ref		ref	
Moderate (175~543)	1.57(1.03-2.39)	0.036	1.59(1.04-2.44)	0.032	1.43(0.91-2.30)	0.125
High (>544)	3.05(2.08-4.55)	<0.001	3.19(2.16-4.79)	<0.001	2.04(1.29-3.24)	0.002
P for trend		<0.001		<0.001		0.002

Model 1: unadjusted **Model 2**: adjusted for age, gender, race; **Model 3**: adjusted for age, gender, race, SAPSII score, SOFA score, charlson comorbidity score, CHF, arrhythmias, stroke, COPD, liver, malignancy, CABG, mechanical ventilation use, Spo2, lactate, WBC, hemoglobin, platelet, total bilirubin, AST, BUN, creatinine, chloride, INR; Abbreviations: Coef**.** coefficient;
